# The Status of Maculopathy in Diabetes and Prediabetes Patients in a Population-Based Study Detected by Optical Coherence Tomography: The 2011 Health Examination Survey in Beijing

**DOI:** 10.1155/2017/6513076

**Published:** 2017-07-20

**Authors:** Xi Cao, Zhong Xin, Shiming Li, Yue Qi, Mingxia Yuan, Xiaorong Zhu, Jin-kui Yang

**Affiliations:** ^1^Department of Endocrinology, Beijing Tongren Hospital, Capital Medical University, Beijing 10730, China; ^2^Beijing Key Laboratory of Diabetes Research and Care, Beijing 100730, China; ^3^Beijing Tongren Eye Center, Beijing Tongren Hospital of Capital Medical University, Beijing 100730, China

## Abstract

**Objective:**

The aim of the study was to investigate the prevalence and the risk factors of maculopathy detected by optical coherence tomography (OCT) in a Chinese population with diabetes or prediabetes.* Method*s. 8,155 people were randomly selected to participate in the 2011 annual Health Examination Survey in Beijing. A 75 g oral glucose tolerance test (OGTT) was tested in 3760 subjects with fasting plasma glucose (FPG) ≥ 5.6 mmol/L. Of 3,760 subjects, 583 were also randomly selected to take OCT.

**Results:**

In this study population, 21 (3.95%) patients had maculopathy. Eight patients had diabetes macular edema (DME) and the prevalence was 6.72% in diabetes patients and 1.51% in all subjects. Eleven patients had age-related macular degeneration (AMD) and the prevalence was 3.36% in diabetes patients and 2.07% in all subjects. Logistic regression model confirmed that elevated HbA1c (*p* < 0.001) and systolic pressure (*p* < 0.05) made significant contributions to DME. Stepwise regression analysis revealed that HbA1c and blood creatinine were significantly independent influence factors for central subfield thickness (CST) (*p* = 0.01, *p* < 0.001).

**Conclusions:**

High prevalence of maculopathy was found in patients with diabetes in a Chinese population. Maculopathy poses a significant public health problem in China with rapid rising trend of diabetes.

## 1. Introduction

Type 2 diabetes mellitus (T2DM) has become a major public health problem in China and in the world, affecting 21 million people globally. The prevalence of total diabetes and prediabetes in China has been 9.7% and 15.5% in 2010 [1]. As its prevalence increases, so do the risks of comorbidities, including the risk for eye diseases, such as diabetic retinopathy [2] and diabetic macular edema (DME).

Maculopathy is the main cause of legal blindness in adults aged between 20 and 74 years in industrial nations [3]. Several national and regional studies have documented variable rates of macular diseases in different populations. DME is a major cause of visual loss in patients with diabetes [2]. Yau et al.'s 2012 epidemiological meta-analysis of almost 23,000 subjects worldwide found that DME affected 6.8% of all people with diabetes [4, 5]. Meanwhile, age-related macular degeneration (AMD) is the major cause of blindness among aged populations. In the Republic of Ireland, AMD is estimated to account for 25% of all blind registration (57.1 per 100,000 adults) [6].

Maculopathy has traditionally been assessed by clinical examination, stereoscopic retinal photographs, and fluorescein angiography. Optical coherence tomography (OCT) is a convenient and noninvasive method of imaging the macula. It creates a thickness profile of the retina that mimics quite accurately the histological arrangement of the retina. It is a more objective and accurate method of diagnosing macular edema than clinical examination, even by experts [7]. As the number of people with diabetes is rapidly increasing in China and in the world [1], macular diseases, especially DME, poses significant health benefit. In a survey from Beixinjing District of Shanghai City in China, the status of diabetic retinopathy and DME in patients with T2DM was reported [8]. However, no population-based survey on the prevalence of macular diseases in diabetes and prediabetes of Chinese populations by OCT has been made yet. From a healthcare aspect, data on the prevalence of maculopathy is essential for the future planning of healthcare strategy in this area.

In the present study, we sought to determine the prevalence of maculopathy detected by OCT and investigate possible correlation of maculopathy with systemic risk factors in patients with diabetes and prediabetes in a population-based sample of Beijing residents.

## 2. Materials and Methods

### 2.1. Study Population

From July 2010 to March 2011, we conducted the 2011 Health Examination Survey in Beijing, a cross-sectional, population-based survey on chronic diseases and risk factors. Our study area was Changping, a rapidly developing district in Beijing with an area of 1,343.5 square kilometers and a permanent resident population of 1,660,500. Household sampling was performed by the Center for Disease Control and Prevention (CDC) of Beijing; 8,155 randomly selected households were eligible (occupants were of Chinese ethnicity and had resided in Changping for more than 6 months). All household residents 18–79 years of age were enumerated in each sampled household; then, using Kish's selection tables, one person was randomly selected. Of the 8,155 individuals, 8084 received baseline examinations including physical checks, fasting plasma glucose (FPG) measurements, renal and liver function tests, and a general health questionnaire obtained through face-to-face inquiry. Then, 3,760 subjects with FPG ≥ 5.6 mmol/L were invited to receive a 75 g oral glucose tolerance test (OGTT) and ophthalmic examination. According to the protocol of this study, which is part of the 2011 Health Examination Survey in Beijing, we applied cluster random sampling method to sample subjects. In this process, a random sample (12 villages, 830 subjects) of the total of 48 villages was selected, and then all subjects in each sampled village were included. Finally, 583 subjects (70.2%) out of 830 subjects took part in our OCT study. Of the 1,166 eyes (583 subjects), 119 eyes were excluded for any of the following reasons: (1) self-reported history of trauma or surgery; OCT signal strength < 40 and scans of suboptimal quality with standard deviation of center point of >10% were excluded. For analysis of the correlation of risk factors with CST, only one eye of each patient was used in the study. In the patients who had maculopathy, the eyes with maculopathy were chosen. If both eyes had maculopathy, the right eye was chosen. In the other patients, if both eyes were eligible, the eye with the greater number of OCTs available for review was chosen, and if this number was equal for the two eyes, the right eye was chosen. Thus, a total of 531 eyes (522 right eyes and 9 left eyes) in 531 patients underwent analysis.

The study protocol was approved and monitored by the Ethics Committee of Beijing Tongren Hospital, Capital Medical University, and written informed consent was obtained from all participants at the time of examination.

### 2.2. Laboratory Measurements

Plasma glucose was determined by the glucose oxidase method. Total cholesterol, high-density lipoprotein (HDL) cholesterol, low-density lipoprotein (LDL) cholesterol and triglyceride, urinary albumin, and blood creatinine were analyzed by enzymatic methods on a Hitachi 7600 analyzer using an enzymatic assay.

In a standard 75 g OGTT, blood samples were collected from an antecubital vein after an overnight fast for the determination of plasma glucose and hemoglobin A1c(HbA1c) levels. After the fasting blood specimen had been taken, the OGTT was performed between 08:00 and 10:00 hours. Two hours later, a blood sample was obtained for the determination of postloading plasma glucose levels. All blood samples were sent for centralized analysis within 24 hours.

HbA1c was measured in whole blood by a high-pressure liquid chromatographic assay (VARIANT II, BIO-RAD Lab., Hercules, CA, USA). The normal range for HbA1c was 4.0–6.0%.

### 2.3. Classification of Glucose Homeostasis

FPG of ≥7.0 mmol/L or OGTT-2h plasma glucose (2hPG) of ≥11.1 mmol/L cases were classified as diabetes. Impaired glucose tolerance (IGT) was defined on the basis of FPG < 7.0 mmol/L, 2hPG ≥ 7.8, and <11.1 mmol/L. Impaired fasting glucose (IFG) was defined on the basis of FPG ≥ 6.1 and <7.0 mmol/L and 2hPG of <7.8 mmol/L. Of the total 531 subjects, 234 had normal glucose homeostasis, 178 had prediabetes (IFG and/or IGT), and 119 had diabetes.

### 2.4. OCT Measurements

**Figure 1:**
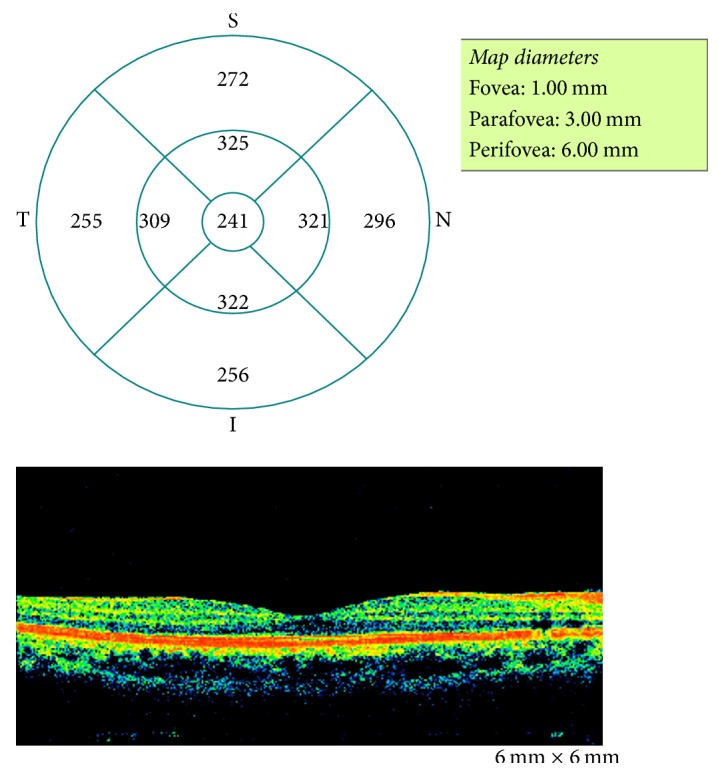
*Representative data sheet of the macular thickness protocol from the OCT*. The macular thickness is measured in 6 mm × 6 mm circles. Central subfield thickness (the thickness of the 1 mm concentric circles (Fovea, 1 mm)) are examined for the clinical studies. These data were obtained from a normal subject.

OCT fast macular scans were obtained by a trained technician from each eye of the participant using 6.0-mm OCT scans (Optovue Inc., Fremont, CA, USA) with software system version 1.5. Fast macular thickness map scan protocol was followed to obtain 6 consecutive macular scans automatically in 3 concentric circles (1 mm, 3 mm, and 6 mm) (Figure 1).

Three scans for each eye were obtained and the best quality one (signal strength index < 40) was selected for analysis. Moreover, one retinal specialist carefully reviewed these scans for abnormalities such as vitreoretinal tractions, retinoschisis, and lamellar macular holes. Retinal thickness over the macula was automatically determined by the instrument software.

### 2.5. Diagnoses of Maculopathy

DME was defined according to Afef Maalej's protocol [9]. It divided an OCT classification of DME into five types: type 1, focal macular thickening; type 2, diffuse macular edema without cysts; type 3, cystoid macular edema; type 4, tractional macular edema; and type 5, serous retinal detachments. This classification is exhaustive exposing the whole aspects of DME.

The people aged 50 years inclusive or older in the present calendar year were included in the AMD study. AMD was defined as dry AMD (drusen (discrete whitish-yellow spots located external to the neuroretina or RPE), drusen with RPE pigmentary abnormalities (areas of hyperpigmentation or hypopigmentation), geographic atrophy of the RPE in the absence of neovascular), and wet AMD (neovascular AMD) [10]. Other maculopathy form was defined as the people aged below 50 years with choroidal neovascularization (CNV).

### 2.6. Statistical Analysis

All statistical analyses were conducted with the software package SPSS version 17 (SPSS Inc., Chicago, IL, USA). Baseline characteristics were expressed as means ± standard deviation for quantitative variables and as percentages for categorical variables. Student's *t*-tests were used to compare means of continuous variables. Chi-squared tests were used to compare proportions. Statistical correlations were obtained via Pearson correlation tests. Logistic regression models were used to detect risk factors in subjects, one for DME and the other for AME. The clinical parameters included in the model were age, gender, systolic BP (SBP), diastolic BP (DBP) and body mass index (BMI), HbA1c, total cholesterol, TG, HDL-C, LDL-C, and blood creatinine. Stepwise regression analysis was used to detect influence factors for central subfield thickness (the thickness of the 1 mm concentric circles (CST)). For all statistical tests, *p* values of less than 0.05 were considered to be statistically significant.

## 3. Results

**Figure 2:**
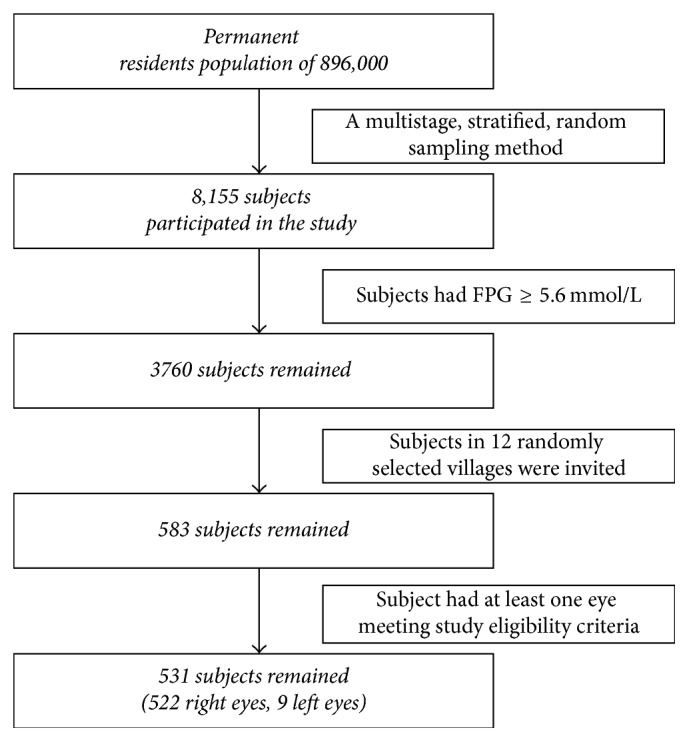
Flowchart of the study.

Table 1 shows demographic characteristics of the patients. The study included 531 subjects who had at least one eye meeting study eligibility criteria (Figure 2). Among them, 311 (59%) were women, and 220 (41%) were men. 119 were found to have had diabetes, 178 had prediabetes, and 234 had normal glucose homeostasis.

In this study population, 21 (3.95%) patients (29 eyes) had maculopathy. Eight patients (8 right eyes, 6 left eyes) had DME and the prevalence was 6.72% in diabetes patients and 1.51% in all subjects. OCT classification of DME in five types: type 1, focal macular thickening (0 eyes, 0%); type 2, diffuse macular edema without cysts (2 eyes, 14.28%); type 3, cystoid macular edema (7 eyes, 50%); type 4, tractional macular edema (3 eyes, 21.43%); type 5, serous retinal detachments (2 eyes, 14.28%). Eleven patients (8 right eyes, 5 left eyes) had AMD, and the prevalence was 3.36% in diabetes patients, 2.07% in all subjects, and 4.55% in the people aged 50 years or older. Additionally, 2 patients (2 left eyes) had other maculopathy forms (Table 2).

Patients with DME rated significantly higher in 2 h PG, HbA1c, and blood pressure compared with no-DME patients (Table 3). In DME patients, there are 3 DMEs with mild DR, 3 DMEs with severe DR, 2 DMEs with PDR (the retinopathy categories used were none (no DR), mild/moderate nonproliferative DR (NPDR), severe nonproliferative, and proliferative DR (PDR) according to ETDRS scale [2]). Logistic regression models confirmed that elevated HbA1c (OR 2.34, 95% CI: 1.64–3.34; *p* < 0.001) and systolic pressure (OR 1.05, 95% CI: 1.01–1.09; *p* < 0.05) made significant contributions to DME. After adjusting for age, gender, BMI, HbA1c, systolic pressure, diastolic pressure, blood creatinine, and lipid, elevated age (OR 1.10, 95% CI: 1.04–1.17; *p* < 0.01) made a significant contribution to AMD.

Correlation of central subfield thickness and the thickness of the 1-mm concentric circles (CST) with different biochemical parameters was also tested. As shown in Table 4, HbA1c, blood creatinine, and blood pressure were significantly correlated with CST. Stepwise regression analysis revealed that HbA1c (*p* = 0.01) and blood creatinine (*p* < 0.001) were significantly independent influence factors for CST (Table 5).

## 4. Discussion

According to the 2011 Health Examination Survey in Beijing, the prevalence of DR in patients with diabetes and prediabetes was 9.9% and 1.2%, respectively [11, 12]. Besides DR, DME is another major cause of vision loss. However, the epidemiology of maculopathy has not been well documented in Chinese populations. This is the first population-based study of maculopathy that includes an OGTT, allowing for identification of all of people with diabetes and prediabetes. It provides new data on the epidemiologic characteristics of maculopathy in Chinese adults.

In Europe, the prevalence of visual impairment due to DME is estimated to be 5.4% of people with diabetes [13]. From a primary source clinical study in the United Kingdom in 2010, an estimated 7.1% of people with diabetes had DME in one or both eyes [14]. In India, of 6,792 people with diabetes, the prevalence of any diabetic retinopathy was 34.1% [15], while 6.4% were found to have DME [16]. In Barbados, DR was present in 28.5% of black/mixed race people with diabetes [17]. Of these patients, less than 1% were found to have proliferative DR but 8.6% were found to have sight-threatening DME [17]. In rural China, one study revealed that the overall prevalence of DME was 5.2% [18]. Our current study showed that the prevalence of DME among patients with diabetes in Beijing was 6.72%—a percentage similar to those found in Barbados [17] and in the United Kingdom [14]. The difference in prevalence between our study (6.72%) and the studies in rural China (5.2%) [18] may be due to the different examination techniques (nonmydriatic retinal camera versus OCT) and disparities in the level of economic development in the two areas with different lifestyles. Regarding the examination techniques, Brown et al. and Browning et al. suggested that OCT is superior to contact lens biomicroscopy for detecting DME, especially in mild cases [19, 20]. Various systemic factors have been associated with increased incidences of DME, such as duration of diabetes [21], levels of albuminuria [15, 21], cholesterol [15, 22], serum creatinine [21], uncontrolled renal parameters, hypertension [15, 23], and aging and genetic defects [24, 25]. Our study confirmed that elevated blood glucose and systolic pressure made significant contributions to DME.

Clinically significant macular edema (CSME) was assigned in cases of the presence of retinal thickening or hard exudates associated with adjacent retinal thickening within 500 *μ*m of the center of the foveal avascular zone or the presence of an area or areas of retinal thickening of at least 1 disc diameter within 1 disc diameter from the center of the macula [26]. Retinal thickness progressively increases from the nonedema group toward the CSME group [27, 28]. Before OCT, slit-lamp biomicroscopy and stereoscopic photography were used to evaluate macular thickening. These methods are relatively insensitive to small changes in retinal thickness and thus insufficient in evaluating structural abnormalities in the retina. OCT can quantitatively measure macular thickness with an axial resolution by up to 7 *μ*m and is more sensitive and objective in detecting the change of macular status than binocular clinical examination. As a consequence, OCT examination should be the first choice to detect possible macular thickening not seen clinically, which has been termed subclinical DME [19]. Sasaki et al. reported that HbA1c, urine protein, and LDL cholesterol were positively associated with CST [29]. Elevated blood pressure also alters the retinal arteriolar hemodynamics, causing a reduction in the compliance of the arteriolar circulation with increasing risk of DME [30]. In our study, HbA1c and blood creatinine were significantly independent influence factors for CST.

Further, there are 2.07% subjects who suffered from AMD and prevalence was 4.55% in the people aged 50 years or older. In the United States, AMD affects 7 million persons 40 years of age and older [31]. China is gradually entering the aging society; AME will also be a public health problem in China. Unfortunately, there is no sufficient recognition of this problem in China. Thus, large-scale and arranged epidemiologic survey and health education are urgently needed.

Our study has several limitations. Firstly, subjects with trauma, surgery, and severe cataracts were excluded from the study, which may have led to an underestimation of the prevalence of maculopathy. Secondly, our results may be bias due to a limited number used in this study. In subsequent investigations, a larger sample size prospective study would be preferable. Thirdly, we did not use the invasive angiography to make a definitive diagnosis of maculopathy in this health examination survey. Finally, it is a cross-sectional study, and we cannot draw conclusions about cause-effect relationship between CST and the metabolic factors.

## 5. Conclusion

In summary, the current study provides novel and vital data on the epidemiologic characteristics of maculopathy in a population-based sample of Chinese adults. DME and AMD are the most important reasons of maculopathy in Chinese population. Considering the high prevalence of diabetes and aging population in China, maculopathy poses a significant public health problem in China. Regular and periodic screening of it should be performed in those people.

## Figures and Tables

**Table 1:** Characteristics of participants with different glucose homeostasis.

	Normal	Prediabetes	Diabetes
Number of subjects	234	178	119
Age (yrs)	45.69 ± 12.09	50.78 ± 10.33	54.90 ± 9.60
Gender			
Female (%)	133 (56.8)	106 (59.6)	72 (60.5)
Male (%)	101 (43.2)	72 (40.4)	47 (39.5)
FPG (per mmol/L)	5.61 ± 0.27	6.30 ± 0.37^*∗*^	8.87 ± 2.64^*∗*^
OGTT-2hPG (per mmol/L)	5.63 ± 1.18	7.23 ± 1.78^*∗*^	14.89 ± 6.01^*∗*^
HbA1c (1%)	5.70 ± 0.37	5.90 ± 0.47^*∗∗*^	7.42 ± 1.80^*∗*^
Total cholesterol (mmol/L)	4.94 ± 0.99	5.09 ± 0.87	5.36 ± 0.88^*∗*^
Triglycerides (mmol/L)	1.78 ± 1.44	1.81 ± 0.96	2.30 ± 1.57
HDL cholesterol (mmol/L)	1.35 ± 0.26	1.40 ± 0.23	1.45 ± 0.25^*∗*^
LDL cholesterol (mmol/L)	2.50 ± 0.49	2.58 ± 0.43	2.70 ± 0.44^*∗*^
Uric acid (*µ*mol/L)	292.94 ± 88.64	291.92 ± 77.17	291.95 ± 79.31
Systolic BP (mmHg)	134.89 ± 19.28	143.28 ± 20.19^*∗*^	148.65 ± 20.13^*∗*^
Diastolic BP (mmHg)	82.66 ± 10.74	86.99 ± 10.34^*∗*^	87.05 ± 11.66^*∗*^
BMI (per kg/m^2^)	25.38 ± 3.46	26.15 ± 3.50^*∗∗*^	27.21 ± 3.49^*∗*^

Values are means (standard deviation) or *n* (%); FPG: fasting plasma glucose; 2hPG: 2-hour postprandial plasma glucose; HbA_1c_: hemoglobin A_1C_; HDL: high-density lipoprotein; LDL: low-density lipoprotein; BMI: body mass index; Student's *t*-test; compared with normal: ^*∗*^*p* < 0.001, ^*∗∗*^*p* < 0.01.

**Table 2:** The prevalence of maculopathy in participants with different glucose homeostasis.

	Normal	Prediabetes	Diabetes	Total
	(*N* = 234)	(*N* = 178)	(*N* = 119)	
DME	0	0	8 (6.72%)	8 (1.51%)
AMD	3 (1.28%)	4 (2.25%)	4 (3.36%)	11 (2.07%)
Other	1 (0.43%)	1 (0.56%)	0	2 (0.38%)
Total	4 (1.71%)	5 (2.81%)	12 (10.08%)	21 (3.95%)

DME: diabetic macular edema; AMD: age-related macular degeneration.

**Table 3:** Characteristics of diabetes participants by DME status.

	No DME	DME
Number of subjects	111	8
Age (yrs)	54.74 ± 9.43	57.08 ± 12.34
Gender (M/F)	45/66	2/6
FPG (mmol/L)	8.74 ± 2.62	10.55 ± 2.37
OGTT-2hPG (mmol/L)	14.36 ± 5.76	22.12 ± 4.77^*∗*^
HbA1c (%)	7.27 ± 1.75	9.53 ± 1.04^*∗*^
Total cholesterol (mmol/L)	5.34 ± 0.88	5.58 ± 0.94
Triglycerides (mmol/L)	2.27 ± 1.40	2.71 ± 3.29
HDL cholesterol (mmol/L)	1.45 ± 0.24	1.44 ± 0.34
LDL cholesterol (mmol/L)	2.67 ± 0.44	2.82 ± 0.47
Creatinine (mmol/L)	74.94 ± 12.99	75.14 ± 13.36
Systolic BP (mmHg)	147.50 ± 19.67	164.50 ± 20.98^*∗∗*^
Diastolic BP (mmHg)	86.89 ± 11.67	89.38 ± 11.51
BMI (kg/m^2^)	27.23 ± 3.56	26.98 ± 2.57

Values are means (standard deviation) or *n* (%); FPG: fasting plasma glucose; 2hPG: 2-hour postprandial plasma glucose; HbA1c: hemoglobin A1c; HDL: high-density lipoprotein; LDL: low-density lipoprotein; BMI: body mass index. Student's *t*-test; compared with normal: ^*∗*^*p* < 0.001, ^*∗∗*^*p* < 0.05.

**Table 4:** Correlation of risk factors with CST.

Risk factor	Mean ± SD	minimum	maximum	*r*
Age (yrs)	49.46 ± 11.57	19	81	0.075
FPG (mmol/L)	6.57 ± 1.80	4.66	17.65	0.023
OGTT-2hPG (mmol/L)	8.22 ± 4.78	2.72	33.47	0.085
HbA1c (%)	6.15 ± 1.15	4.50	13.80	0.099^*∗∗*^
Total cholesterol (mmol/L)	5.08 ± 0.94	2.62	9.63	0.012
Triglycerides (mmol/L)	1.91 ± 1.35	0.40	12.5	0.025
HDL cholesterol (mmol/L)	1.39 ± 0.25	0.72	2.30	0.013
LDL cholesterol (mmol/L)	2.57 ± 0.46	1.32	4.45	0.013
Creatinine (mmol/L)	76.76 ± 15.42	44.20	212.16	0.178^*∗*^
Systolic BP (mmHg)	140.78 ± 20.52	97.50	220.00	0.106^*∗∗*^
Diastolic BP (mmHg)	85.09 ± 11.01	60.00	127.20	0.092^*∗∗*^
BMI (kg/m^2^)	26.05 ± 3.55	18.08	43.71	0.069

FPG: fasting plasma glucose; 2hPG: 2-hour postprandial plasma glucose; HbA1C: hemoglobin A1C; HDL: high-density lipoprotein; LDL: low-density lipoprotein; BMI: body mass index; Pearson's correlation test. ^*∗*^*p* < 0.001, ^*∗∗*^*p* < 0.05.

**Table 5:** CST associated factors based on stepwise regression analysis.

	*β*	SE	*t*	*p*
Creatinine (*µ*mol/L)	0.302	0.071	4.240	<0.001
HbA1c (%)	2.462	0.950	2.592	0.01

HbA1C: hemoglobin A1C; the clinical parameters included in the stepwise regression analysis were age, systolic BP (SBP), diastolic BP (DBP), body mass index (BMI), HbA1c, total cholesterol, triglycerides, HDL-C, LDL-C, and blood creatinine.

## Additional Points

*Highlights*. (1) This study provides new data on the epidemiologic characteristics of maculopathy in a population-based sample of Chinese adults. (2) Diabetic macular edema (DME) and age-related macular degeneration (AMD) were the most important reasons of maculopathy in Chinese population. (3) Considering the high prevalence of diabetes and aging population in China, maculopathy poses a significant public health problem in China; regular and periodic screening should be considered in those people.
